# Photonic Crystal Microchip Laser

**DOI:** 10.1038/srep34173

**Published:** 2016-09-29

**Authors:** Darius Gailevicius, Volodymyr Koliadenko, Vytautas Purlys, Martynas Peckus, Victor Taranenko, Kestutis Staliunas

**Affiliations:** 1 Laser Research Center, Department of Quantum Electronics, Vilnius University, Sauletekio Ave. 10, LT-10222, Vilnius, Lithuania; 2 International center “Institute of Applied Optics” NAS of Ukraine, Kudryavskaya Str. 10G, 04053, Kyiv, Ukraine; 3 Departament de Física i Enginyeria Nuclear, Universitat Politècnica de Catalunya, Colom 11, 08222 Terrassa, Spain; 4 Institucio Catalana de Reserca i Estudis Avançats (ICREA), passeig Lluis Companys 23, 08010 Barcelona, Spain

## Abstract

The microchip lasers, being very compact and efficient sources of coherent light, suffer from one serious drawback: low spatial quality of the beam strongly reducing the brightness of emitted radiation. Attempts to improve the beam quality, such as pump-beam guiding, external feedback, either strongly reduce the emission power, or drastically increase the size and complexity of the lasers. Here it is proposed that specially designed photonic crystal in the cavity of a microchip laser, can significantly improve the beam quality. Experiments show that a microchip laser, due to spatial filtering functionality of intracavity photonic crystal, improves the beam quality factor M^2^ reducing it by a factor of 2, and increase the brightness of radiation by a factor of 3. This comprises a new kind of laser, the “photonic crystal microchip laser”, a very compact and efficient light source emitting high spatial quality high brightness radiation.

The microchip lasers (and generally other micro- and millimeter size lasers such as edge-emitting or vertically-emitting semiconductor lasers) usually suffer from a low beam quality, especially in high power operation regimes. There have been proposed several techniques to improve the beam quality of microchip lasers, e.g. by using optical injection^1^, external feedback^2^, external gratings^3^,^4^, external beam manipulation techniques^5^ and others, however, this results in a loss of the main advantage–the compactness and simplicity of the laser design.

In order to obtain high beam quality in microchip lasers usually the emission area is confined in transverse space, localizing the fields by a defect^6^ or by using a relatively narrow pump area. The restriction of the pump area and pump intensity enables a single-transverse mode emission, however, strongly reduces the emitted power (*P*_*out*_). The increase of the pump power (either by increasing pump area or the pump intensity) results in multi-transverse mode emission, and therefore in a drastic reduction of the beam quality. The power of emitted radiation still increases with the increase of the pump power, however, the brightness (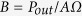, where *A* is the surface area of the beam cross-section, *Ω* is the solid angle of divergence) of the radiation saturates and does not increase.

Our idea, elaborated theoretically and proved experimentally in this letter, is that the specially designed intracavity Photonic Crystals (PhC) can solve the beam quality problem **for high pump power** decreasing the beam quality factor M^2^. Photonic crystals, besides their celebrated properties in the frequency domain, such as frequency bandgaps and slow light effects^4^,^7^,^8^, can also affect the spatial beam propagation, causing such peculiarities as the negative refraction^9^,^10^, anomalous diffraction (flat lensing)^11^,^12^, and importantly for the present work–spatial (angular) filtering. The spatial filtering by PhCs, is based on the angular band-gaps PhCs^13^,^14^ and angular quasi-bandgaps PhCs^15^,^16^, and does not require the access to the far field domain of resonating field like in conventional filtering techniques by confocal lens arrangement (see Supplementary Information for detailed description of PhC spatial filtering). The PhC spatial filters can provide efficient filtering in extremely short propagation distances (typically around 200 μm)^17^. The arrangement, therefore, comprises an efficient, compact laser source, which we named the microchip photonic crystal laser, able to emit powerful beams of good spatial quality (single transverse mode beams), and of high brightness.

Being specific, we consider a Nd: YAG based microchip laser, of cavity length 5 mm, and maximum emission power 900 mW (in multi-transverse mode regime). To ensure single transverse mode emission, the width of the pump beam is restricted (typically to 80 μm, depending on pump power). The use of broader pump area increases the Fresnel number, and results in multimode emission if no special means are taken. We propose to use the spatial PhC filters inside the cavity, in order to maintain a high beam quality while increasing the pump area and pump intensity.

For that purpose we fabricated PhC filters of two different symmetries: the axisymmetric filter^18^ (Fig. 1d) to achieve the main goal, i.e. the single-transverse mode emission; and also a one-dimensional (1D) spatial filter^17^ (Fig. 1a), filtering in one transverse direction. The latter allows to convincingly demonstrate the appearance of a beneficial filtering effect, i.e. to have simultaneously a laser with- and without filtering in different directions in transverse space under identical conditions (the same pump power and the same losses for both field quadratures). We fabricated the PhCs using point-by-point direct laser writing technique, see the ***Fabrication method***. The ratio between the longitudinal and transverse periods of the index modulation in the photonic crystal structure provides the angular position of the filtering range. For instance, the high angle pass filtering (removal of the axial mode) is obtained for 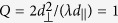 (see Supplementary Information for the calculation of the filtering angles in passive configuration). In order to obtain the low-angle pass filtering (transparent for the axial mode and opaque for small angle of-axis modes), which is the purpose of our work, the geometry parameter *Q* slightly deviates from unity.

The PhC filters were first tested in stand-alone configuration (see the experimentally measured transmission in Fig. 1c,f, also Supplementary Information), and then positioned inside the cavity of microchip laser. The experimental outcome is shown in Fig. 1h. The axisymmetric spatial filter in the cavity of microchip laser causes the narrowing of the far field radiation (decrease of angular spread of the beam) and formation of single-transverse-mode beam. For comparison 1D filter results in the decrease of the angular divergence along the filtering direction only (vertical direction in Fig. 1g) whereas the beam in unaffected (horizontal) direction remains largely diverging (containing higher order transverse modes).

Figure 2 summarizes the quantitative experimental results. Without the PhC filter the increasing pump power results in the increase of the emission power and of the divergence of the output beam, whereas the brightness of the radiation remains approximately constant. With the PhC filter in microcavity the divergence of the beam remains independent on the pump power (up to a particular pump level), and the brightness constantly increases with the pump. Note that the emission power in the presence of the PhC spatial filter just slightly decreases compared to the case without spatial filtering. The increase of brightness due to intracavity photonic crystal is 2.8 (maximum brightness)—3.1 times (at equal pumping) in this particular experimental measurement. Behind a particular threshold for the pump power the spatial filtering is no more sufficient, and the beam quality as well as brightness starts decreasing. Maximal power of single transverse mode beam was 88 mW without filtering and 335 mW with the PhC filter (area with divergence <9 mrad on Fig. 2). It depends on the efficiency of the spatial filtering (the width and the depth of filtered out area in angular domain). Speaking in numbers, the spatial quality factor of the beam from the PhC microchip laser remained M^2^ = 1.2, for the emission power up to 300 mW, whereas the spatial quality factor of the beam from the laser without spatial filtering increased until M^2^ = 2.5 for the same output power^19^.

In order to justify the experimental observations, and also to show the potential of a photonic crystal microchip laser, we performed numerical simulations of the microchip laser with- and without the intracavity spatial filtering. The simulations are based on Maxwell-Bloch model, see ***Numerical methods***. The results are summarized in Fig. 3, where series of calculation results are presented: one series (shown by red lines) approximately corresponds to the current experimental situation, and the other two series corresponding to the spatial filters of 2 and 4 times higher efficiency (beyond what our current technologies allow for today). The far field patterns without and with spatial filtering show the same qualitative features as in experiment (compare with Fig. 2). The qualitative characteristics of numerical simulations (dependence of power, divergence, and brightness of the emitted beam) are also well compatible with experimental observations in Fig. 2.

Note, that the inset in Fig. 3b (the averaged far field at the normalized pump intensity *D*_0_ = 6) shows a dark ring. This happens when the PhC filtering efficiency is no more sufficient to ensure the single-transverse-mode emission. The PhC microchip laser starts emitting at the angles larger than the filtering area. This illustrates the limits of the applicability of spatial filtering for given filtering efficiency. The use of more efficient spatial filters, i.e. with a deeper/broader filtering angular range, would allow increasing the brightness of emitted radiation proportionally, as the Fig. 3b demonstrates.

**Concluding**, we show a substantial improvement of the spatial quality of the beam emitted by microchip laser due to the spatial filtering functionality of intracavity photonic crystals. Specifically, we were able to increase the power of single transverse mode emission from 90 mW without intracavity PhC, to 340 mW with intracavity PhC, i.e. almost 4 times. The brightness of the emitted radiation has been increased approximately by a factor of 3.

The performance can be strongly improved by advancing the angular transmission characteristics of the spatial filters. The angular filtering range is limited by the material characteristics of the filter (the amplitude of refraction index modulation, and the length of the PhC (the number of longitudinal periods). The performance of photonic crystal microchip laser is thus restricted by technological limitation (aberrations of femtosecond laser writing arrangement, available materials) of the PhC filters. With the future advance of microfabrication technologies and with new materials providing the larger refraction index modulation, the performance of the PhC microchip laser can be significantly improved. Technically, the idea can be brought to perfection by fabricating the PhC directly on the microchip active media using DLW techniques, resulting in a completely monolithic device^20^. The present work demonstrates the principle of intracavity spatial filtering by PhCs, and thus constitutes a new class of laser - the photonic crystal microchip laser. The numerical simulations shows huge potential of the photonic crystal microchip laser improved using advanced technologies. In particular, our numerics (not presented here) show that the concept is applicable for pulsed microchip lasers too (by numerically solving MBE Equations 1 with added saturable absorber).

Finally the proposed idea could be applied to the other types of microlasers, like semiconductor edge-emitting lasers, and vertical cavity semiconductor lasers, the VCSEL (in fact the VCSELS would convert into VECSELS, the Vertical ***External*** Cavity Semiconductor Lasers, when one incorporates at approximately 200 μm length PhC filter inside the cavity).

## Fabrication Method

We fabricated the PhCs using point-by-point direct laser writing technique (see Supplementary Information) in the bulk of standard soda-lime microscope glass slides (*n*_*ref*_ = 1.52) using direct laser writing technique. A femtosecond Yb:KGW laser beam (295 fs pulse duration at FWHM) was tightly focused into the sample using 40 × 0.95 NA microscope objective, and producing local modified refractive index areas at the vicinity of the focal point. Thus, by scanning the sample in respect to the focus, 3D PhCs are created. In particular, we used fundamental 1030 nm laser wavelength, 1 kHz pulse repetition rate, 432 nJ energy (246 TW/cm^2^ peak intensity), and 800 μm/s writing speed.

**Figure 1:**
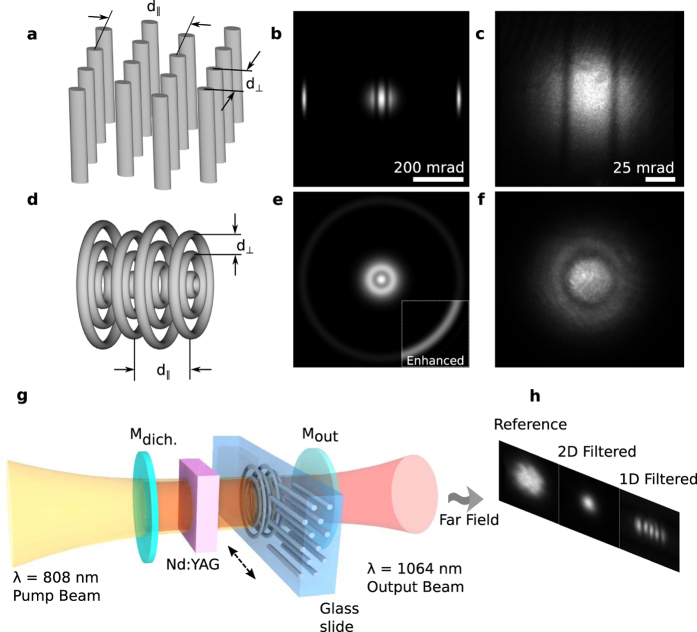
Filtering by one-dimensional and axisymmetric PhC filters. (**a**) Spatial PhC filter, consisting of periodic structure of parallel rods, results in one-dimensional filtering, as shown in single-pass angular far-field distribution, numerically (**b)** and experimentally (**c)**. Respectively, PhCs of circular geometry (**d)** causes axisymmetric filtering (**e**,**f)**. The (**g)** schematically shows the microchip laser structure, with inserted intracavity PhC filters (1D, axisymmetric and no filter), and (**h)** shows the experimentally measured far-field profiles in corresponding cases.

The ratio between the longitudinal and transverse periods of the index modulation in the photonic crystal structure provides the angular position of the filtering range. For instance, the high angle pass filtering (removal of the axial mode) is obtained for 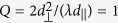 (see Supplementary Information for the calculation of the filtering angles in passive configuration). In order to obtain the low-angle pass filtering (transparent for the axial mode and opaque for small angle of-axis modes), which is the purpose of our work, the geometry parameter *Q* slightly deviates from unity. The transverse period was *d*_⊥_ = 2 μm, and longitudinal period was 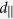 = 9.4 μm, corresponding to a geometry coefficient *Q* ≈ 1.18 for a wavelength of *λ* = 1064 nm. The dimensions of PhC filters were restricted to fabrication time and the focal length of the objective. The lateral dimensions of both 1D and axisymmetric PhC filters were 200 μm, and the length was 188 μm, which corresponds to 40 layers or 20 periods. Each layer consists of parallel rod or concentric phase gratings, having an inverted pattern in respect to the neighboring layers, as conceptually shown in Fig. 1a,d.

We estimated the resulting scattering coefficient to be *s* ≈ 0.025, by calibrating experimentally observed spatial transmittance spectra with numerically derived ones as in ref. 17. This value corresponds to about *s*^2^ ≈ 0.6% integral intensity scattering from one PhC layer.

### Numerical methods

In order to justify the experimental observations, and also to show the potential of a photonic crystal microchip laser we performed numerical simulations of the microchip laser with- and without the intracavity spatial filtering. We numerically integrated the Maxwell-Bloch Equation (MBE) system:


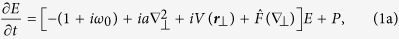



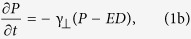



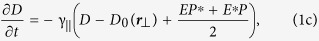


where the envelope of the beam *E*(***r***_⊥_, *t*), the field polarization *P*(***r***_⊥_, *t*), and the population inversion *D*(***r***_⊥_, *t*) are defined in two-dimensional transverse space **r**_⊥_, and evolving in time. *D*_0_(**r**) is the normalized pump profiled. Time is normalized to photon life time 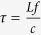, 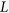 being the full (round-trip) length of the resonator, and *f* is the cavity fines (in experiment *τ* ≈ 0.1 ns). Diffraction coefficient 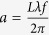 scales the transverse space (in experiment *a* ≈ 2 × 10^3^ μm^2^. 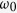 is the resonance detuning from the gain line center, *γ*_⊥_is the polarization relaxation rate, which is typically of order of 10, and 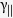 is the inversion decay rate, typically of order of 10^−6^ (both normalized to photon relaxation rate). *V*(***r***_⊥_) is the confining potential in transverse space (in microchip laser we considered thermal lensing, where refraction index follows pump profile *V*(***r***_⊥_) = *α *· *D*_0_(***r***_⊥_)). The filtering function of intracavity PhC is introduced phenomenologically by the operator 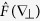 providing the far-field transmission profile 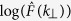, and matching the experimentally measured angular transmission dependences of PhC filters.

## Additional Information

**How to cite this article**: Gailevicius, D. *et al*. Photonic Crystal Microchip Laser. *Sci. Rep.* **6**, 34173; doi: 10.1038/srep34173 (2016).

## Supplementary Material

Supplementary Information

## Figures and Tables

**Figure 2:**
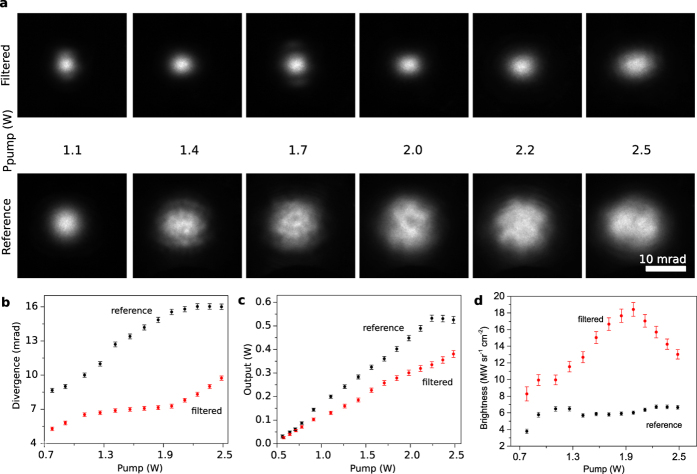
Experimental results. (**a)** Experimentally measured far-field distributions from the microchip laser without a PhC spatial filter (first column), and with (second column) axisymmetric intracavity spatial filter (Q = 1.18, N = 20). The width of the pump beam was 200 μm. Presence of the axisymmetric filter results in TEM00 mode of emitted radiation. (**b**–**d)** shows the beam divergence, output power, and brightness dependencies on the pump power respectively.

**Figure 3:**
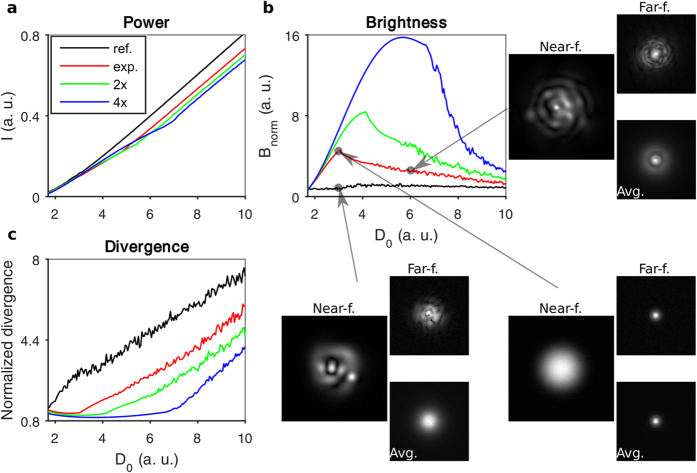
Numerical study. Summary of numerical simulations of photonic crystal microchip laser: (**a**) shows the emission power, (**b**) the brightness and (**c**) the angular divergence of the emitted beam, depending on the pump power, for three realizations of spatial filters. The black line depicts the reference case (no filter), the red line corresponds to the filter with transmission profile corresponding to the experimental situation (Figs 1 and 2), and the green and blue lines correspond to the filters with 2 and 4 times increased filtering performance (correspondingly increased width of the filtering area). The near- and far-field beam profiles as well as averaged far-field profiles are shown on insets: the near field window size is 2 × 2 mm, and far field windows size is 40 × 40 mrad.
