# Supplementary material for: Photonic Crystal Microchip Laser
Source: Sci Rep. 2016 Sep 29;6:34173. doi: 10.1038/srep34173 (PMC5040951; doi:10.1038/srep34173)
Supplement: Supplementary Information [file srep34173-s1.pdf]

Supplementary information:
Photonic Crystal Microchip Laser

Darius Gailevicius*[1], Volodymyr Koliadenko[2], Vytautas Purlys[1], Martynas Peckus[1], Victor Taranenko[2], and Kestutis Staliunas[3,4]

[1]Laser Research Center, Department of Quantum Electronics, Vilnius University, Sauletekio Ave. 10, LT-10222, Vilnius (Lithuania)

[2]International center «Institute of Applied Optics» NAS of Ukraine, Kudryavskaya Str. 10G, 04053, Kyiv (Ukraine)

[3]Departament de Física i Enginyeria Nuclear, Universitat Politècnica de Catalunya, Colom 11, 08222 Terrassa, Spain

[4]Institucio Catalana de Reserca i Estudis Avançats (ICREA), passeig Lluis Companys 23, 08010 Barcelona, Spain

Introduction. For the main article most important are the geometrical characteristics of the Photonic Crystal (PhC) spatial filter, also the estimation of filtering efficiency (the width and the depth of the dip in the angular transmission curve). Below we summarize these two aspects of PhC spatial filtering in a stand-alone (single transmission) configuration, and also estimate that higher refraction index modulation of the PhCs can improve the spatial filtering efficiency.

We also add a detailed description of fabrication procedure of PhC filters, as well as the detailed description of the parameters of microchip laser used.

Geometry. Analytical estimations and calculations of the geometry and efficiency have been performed in 1D filtering configuration, using 2D photonic crystals (see Fig.S1.), however, the estimations work in the 2D case, for axisymmetric filtering (we also compare these two cases by numerical simulations).

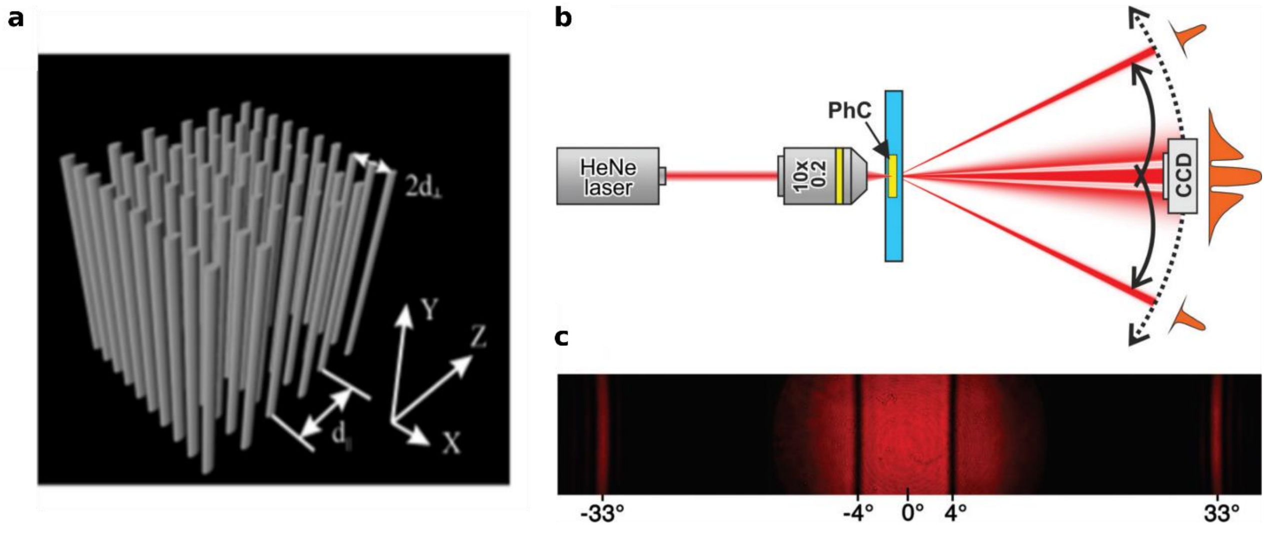

Figure S1. Stand-alone spatial filtering in a 2D PhC.
***a** 2D PhC; **b** filtering experiment; and **c** typical far field angular transmission spectrum.*

The principle of PhC spatial filtering (see also recent review [1]), is based on the angular bandgaps (Fig. S2.a,b)) or angular quasi-bandgaps (Fig. S2.c,d). PhC filtering had been first proposed [2-4] to occur due to the presence of angular bandgaps in Bragg configuration (when the diffracted radiation propagates

backwards); however, the realization is problematic in visible and near-infrared optics, as the Bragg regime requires small longitudinal period (between λ/2 and λ), which is nowadays technically unfeasible. Alternatively, spatial filtering in the Laue configuration was proposed in [5], and later experimentally demonstrated [6,7]. In the latter case of Laue configuration the diffracted radiation propagates forwards, therefore the longitudinal modulation period can be larger than the wavelength. This is an important condition lifting restrictions for the fabrication of PhC filters, especially in visible and near infrared regimes.

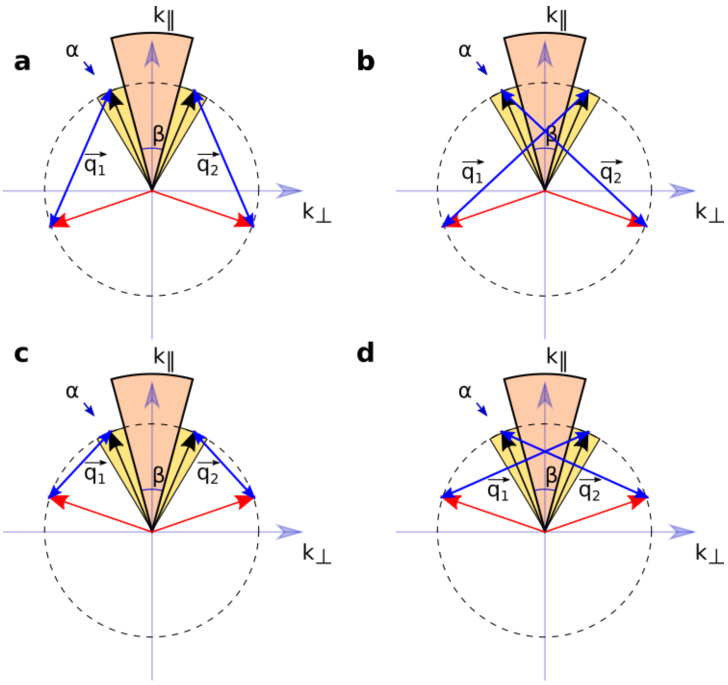

Figure S2. Illustration of the spatial filtering by a 2D PhC, in spatial Fourier domain (k_x , k_y).
In configuration with angular bandgaps (Bragg configuration) ***a***, ***b***, *angular quasi-bandgaps* ***c***, ***d***. *spatial filtering occurs when the waves in the dark triangle zones get into resonant scattering condition via refraction index modulation with wave vectors $q = (q_x,q_z)$ (blue arrows). Thick red arrows show the direction of filtered out angular components. The spatial spectrum (far field) of the initial beam, consisting of the central (regular) part, and of the wings (the part to be removed), is indicated by bright and dark triangles.*

Here we discuss the 1D filtering, but the main principles are valid for 2D axisymmetric filtering [8].

For most applications low-angle-pass filtering is important, in which, in the ideal case, on-axis radiation is 100% transmitted and off-axis components are deflected, i.e. filtered out. The filtering angles depend on the interplay between the longitudinal and transverse periods of the modulation (i.e.: at least 2D modulation is required). In paraxial approximation, the filtering angle obeys the simple relation (which follows from the resonance conditions of modulation- and light wavevectors):

$$\sin(\alpha) = \frac{q_\perp}{2k_0}(Q-1) = \frac{\lambda}{2d_\perp}(Q-1). \qquad \text{(S1)}$$

Here, an adimensional parameter $Q = 2\frac{d_\perp^2}{\lambda d_\parallel}$ depends upon the geometry of the PhC. The relation (S1) shows that at $Q = 1$, the on-axis radiation is filtered out. For Q slightly larger/smaller than unity off-axis radiation at some small angles is filtered out, and on axis radiation can transmit. This dependence as calculated numerically (paraxial approximation) is shown in Fig. S3, where the angular profiles of the transmitted radiation are represented with respect to the adimensional geometry parameter Q. The transmission character is similar for both cases of 1D and 2D-axisymmetric filtering, except for some discrepancies close to $Q = 1$ (the anomalous field concentration on axis, the so called magic spot, predicted in [9] in 2D case).

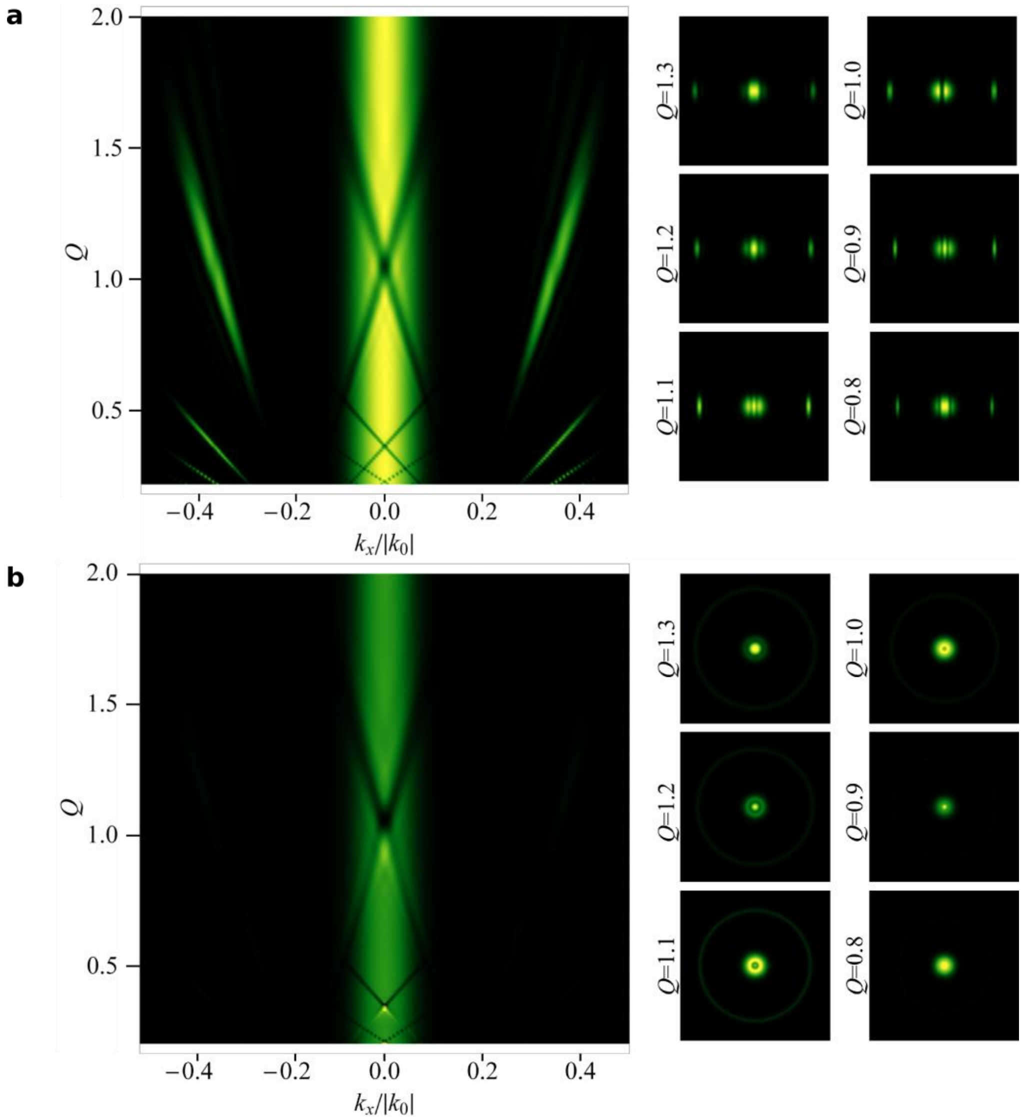

Figure S3. Single pass angular filtering.
*By a one-dimensional PhC **a**, and by an axisymmetric PhC **b** for varying values of geometry factor Q. On the left one dimensional cross-sections of the far-field beam intensity are shown, whereby on the right two-dimensional cross-section are displayed. The PhC parameters are N = 12, s = 0.075 for both cases.*

The filtering characteristics predicted by theory/numerics (Fig. S3) have been measured experimentally in a single pass configuration. Fig. S4 shows the transmission of a Gaussian beams through the axisymmetric PhC filters with varying Q parameter.

Filtering efficiency. While the filtering angles are defined solely by the geometry of the refraction index modulation of the PhC (see Fig.S3-S4), the depth and the width of the dip in angular filtering line depend on two essential parameters: the modulation of the refractive index Δn, and the length of the filter L (or alternatively, the number of the modulation periods along the beam propagation direction N ($L = N \cdot d_{\parallel}$)). Essentially, the factor $f = L \cdot \Delta n/\lambda$ denotes the filtering efficience. To obtain 100% filtering exactly at the central filtering angle (i.e. to achieve that the zero transmission at filtering angle) the factor f, must reach $f = 2$. For larger values ($f > 2$), the filtering performance again decreases. In Fig. S5 are shown examples of single pass filtering with different factor f: too small, optimum, and too large.

The full width of the filtered angular area (the dip in transmission curve) at 100% filtering is approximately: $\Delta\varphi \approx \Delta n \cdot \mathrm{d}_{\perp}/\lambda$ (in radians) (Precisely $\Delta\varphi = \sqrt{3}/2 \cdot \Delta n \cdot \mathrm{d}_{\perp}/\lambda$ in the limit of small Δn).

For instance, for the PhC with a depth of periodic modulation of refraction index $\Delta n = 0.003$ (for ~6.5 μm longitudinal period), the 100% filtering requires appr. 130 μm length of the filter, and the transmission dip at 100% filtering has a width of 1.4 degrees.

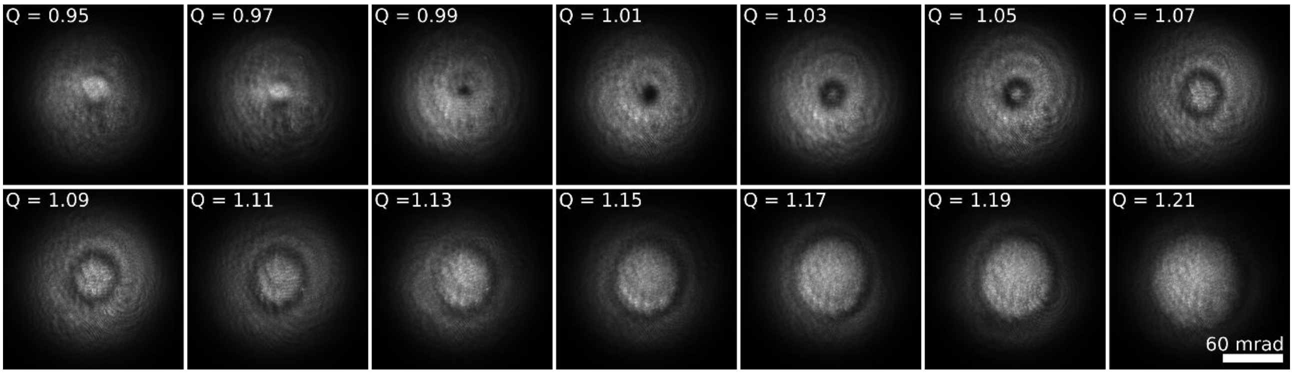

Figure S4. Experimental filtering angle dependence on Q.
Experimentally measured profiles, showing the filtering angle dependence on the geometry factor Q. Different filter configurations are possible for different divergence reduction requirements.

A convenient parameter to characterize the spatial filtering is the diffractive scattering by one modulated period of the filter. Approximating the index modulation by harmonic functions the following value of the diffractive scattering is obtained $s = \pi^{3/2}\Delta n \cdot \mathrm{d}_z/\lambda$. The experimentally obtained values of s were estimated from callibration of experimentally measured transmission function with the numerically calculated one. The filters at optimum fabrication condition show $s = 0.025$, which correspond to $\Delta n = 0.002$.
Important to note is that the 100% filtering efficiency is not necessary for intracavity use of the PhC spatial filters. For a given finesse of the resonators *F* the effective life time of the photon is *F* roundtrips, and the radiation effectively passes through the filter *2F* times. Therefore instead of a narrow line of 100% filtering, a broader, but not so deep dip in the angular transmission profile is more usefull. This, for instance, can be obtained using the chirped PhC filter (see [7] for 1D filtering, and [10] for 2D filtering by chirped PhC crystals). Fig.S5 illustrates the chirped 2D crystal, where the longitudinal period smoothly changes along the structure. The filtering angle respectively sweeps along the structure too (see the equation S1 for filteroing angle depending on longitudinal period), which results in shallower but broader dip in the angular transmission curve.

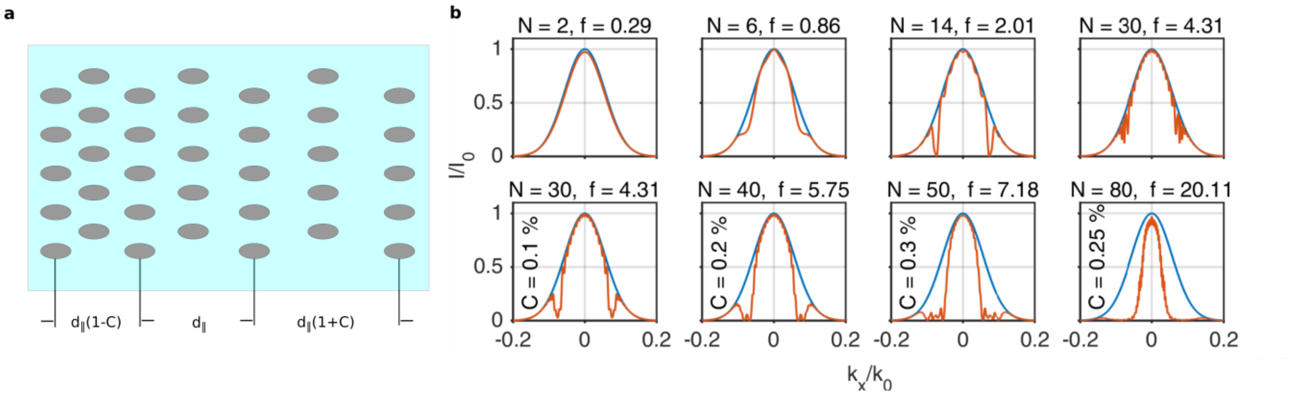

Figure S5. ***Single pass angular filtering of a Gaussian beam by chirped and unchirped PhC.***
a *The concept of chirping of periodic of a PhC structure.* ***b*** *shows the optimum filtering (deepest dip) at* $f = 2$. *For larger f values the filtering efficiency reduces. The chirped structure is efficient for larger values of f, if the f and chirp parameter C are well matched.*

Fabrication of PhC Spatial Filters. PhC filters were fabricated by the direct laser writing (DLW) technology. The DLW technology is based on point-by-point laser modification of a glass sample by tightly focusing a femtosecond laser beam inside the bulk of it. Normally the glass is transparent and the laser

irradiation does not affect it, but at the focal point the intensity is so high that nonlinear ionization occurs, leading to, in this case, a permanent modification of the refractive index of the sample. The size of modified area lies in the submicrometer scale and the precision is mainly determined by the positioning stages.

In our fabrication setup (Fig. S6.a) the samples are positioned by using precise linear stages, having 110x110x60 mm fabrication area and <10 nm positioning errors, and galvanometric mirrors that enable quick DLW beam translation movement without considerable inertia. The PhCs in our case were written in a glass medium. We used standard microscope slides (ISO 8037/1), with a thickness of 1 mm, made from soda-lime glass with a standard composition of SiO_2 72.2 %, Al_2O_3 1.2 %, Fe_2O_3 0.03 %. The slides are placed on the linear translation stages and iteratively displaced in the beam propagation direction, as each in-plain layer is formed by scanning the beam with the galvanometric scanner.

The 1D PhC filters are formed using a simple 3D raster scan, and the axisymmetric PhC (Fig. S6.b,c) filters are formed by writing concentric rings from smallest radius to largest, layer by layer. The axisymmetric filters feature a defect due to the circle start and end point matching. Also, there is some mismatch of the individual layer thickness as shown in Fig. S6.d, due to spherical aberrations.

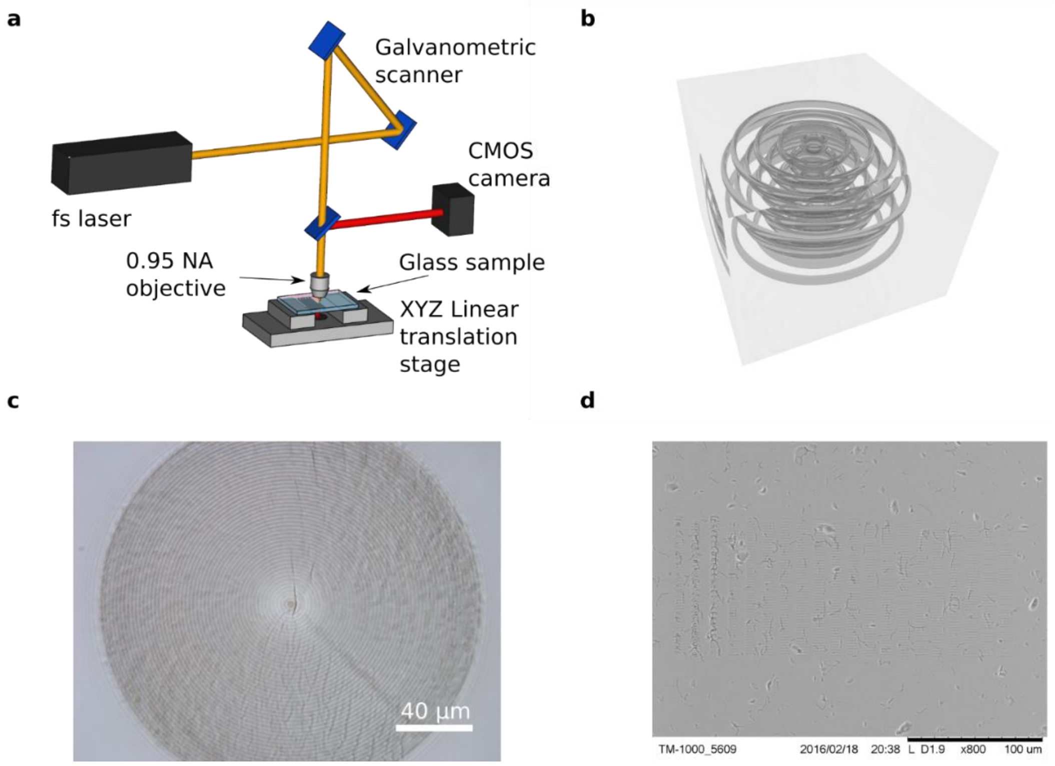

Figure S6. Fabrication and structure of the PhC.

a A *simplified fabrication setup: a glass sample is moved with respect to the tightly focused femtosecond pulse laser beam, resulting in a point-by-point refractive index modification in the bulk of the glass.* ***b*** *A model of the axisymmetric PhC embedded in glass,* **c** *An optical microscopy image of a fabricated photonic crystal (top surface). The transverse period is 2 µm.* **d** *A SEM image of a 2D PhC that has been cleaved and wet etched in a 8M KOH solution (3h, 70 °C, agitated).*

Microchip laser. In the experiment we used an end-pumped microchip Nd:YAG ceramic laser operating in continuous wave regime. A 808 nm fiber-coupled laser diode was used as a pumping source. Through a lens system the pumping beam is focused onto the gain medium with a spot diameter ranged from 80 µm to 200 µm. The gain medium was a plane-parallel bonded YAG/Nd:YAG ceramics with 1.5 mm/1.5 mm thickness of its parts and 2% of Nd doping concentration. The surface of YAG part was coated for high transmission at 808 nm and high reflection at 1064 nm. The other surface of Nd:YAG part was antireflection coated at 1064 nm. A flat mirror with reflection coefficient 94 % at 1064 nm was used as an output coupler.

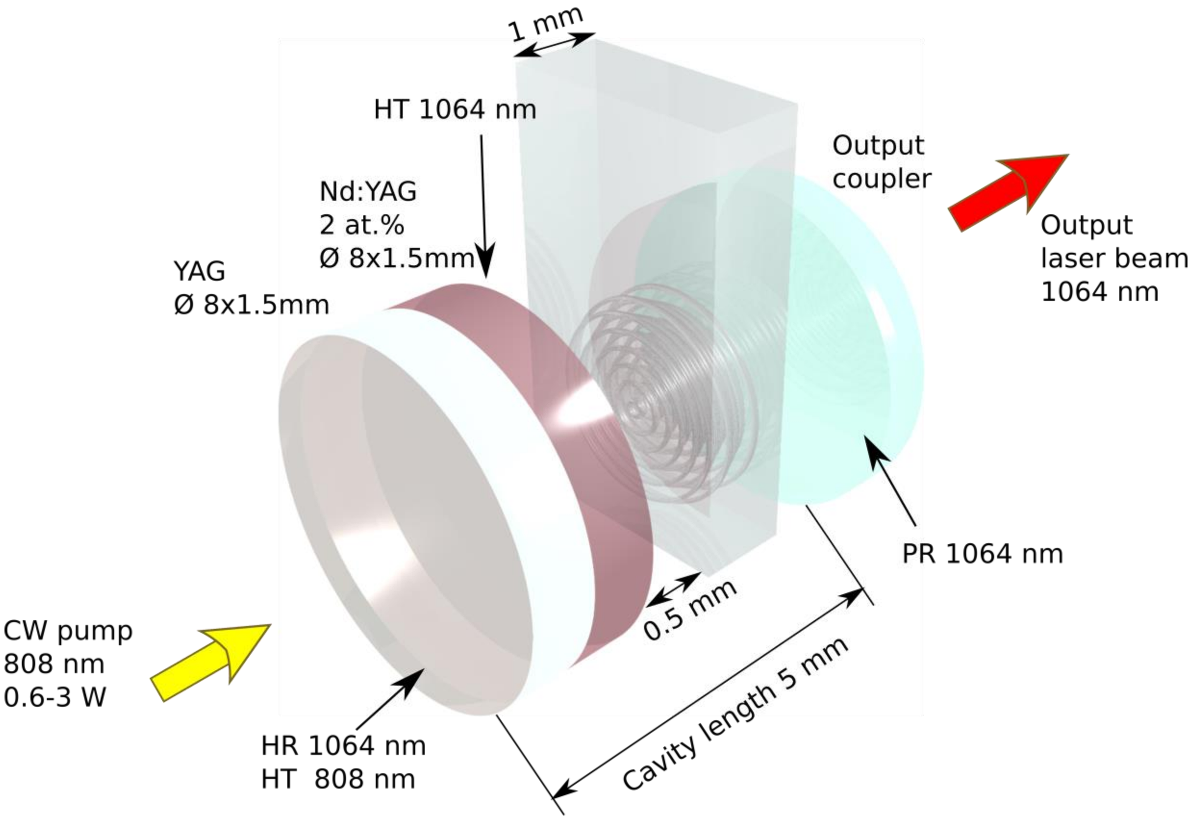

Figure S7. *The detailed configuration of the microchip laser, showing the descriptive parameters.*

References:

1. Maigyte, L. & Staliunas, K. Spatial filtering with photonic crystals. *Appl. Phys. Rev.* **2,** 011102 (2015).
2. Serebryannikov, a. E., Petrov, a. Y. & Ozbay, E. Toward photonic crystal based spatial filters with wide angle ranges of total transmission. *Appl. Phys. Lett.* **94,** 26–29 (2009).
3. Tang, Z., Fan, D., Wen, S. & Zhao, C. Low-pass spatial filtering using a two-dimensional self-collimating photonic crystal. *Chinese Opt. Lett.* **5,** 211–213 (2007).
4. Serebryannikov, A. E., Lalanne, P., Petrov, A. Yu., & Ozbay, E. Wideangle reflection mode spatial filtering and splitting with photonic crystal gratings and single-layer rod gratings. *Opt. Lett.* **39**, 6193 (2014).
5. Staliunas, K., & Sanchez-Morcillo, V. Spatial filtering of light by chirped photonic crystals. *Phys. Rev. A* **79**, 053807 (2009).
6. Maigyte, L. et al. Signatures of light-beam spatial filtering in a three-dimensional photonic crystal. *Phys. Rev. A* **82**, 043819 (2010).
7. Purlys, V. et al. Spatial filtering by chirped photonic crystals. *Phys. Rev. A* **87**, 033805 (2013).
8. Purlys, V. et al. Spatial filtering by axisymmetric photonic microstructures. *Opt. Lett.* **39**, 929–32 (2014).
9. Purlys, V. et al. Super-collimation by axisymmetric photonic crystals. *Appl. Phys. Lett.* **104**, 221108 (2014).
10. Gailevicius, D., Purlys, V., Maigyte, L., Peckus, M., & Staliunas, K. Chirped axisymmetric micro-photonic structures for spatial filtering. *J Nanophotonics* **8**, 084094 (2014).
